# “Connectedness to Nature Scale”: Validity and Reliability in the French Context

**DOI:** 10.3389/fpsyg.2017.02180

**Published:** 2017-12-12

**Authors:** Oscar Navarro, Pablo Olivos, Ghozlane Fleury-Bahi

**Affiliations:** ^1^Faculty of Psychology, University of Nantes, Nantes, France; ^2^Department of Psychology, University of Castilla-La Mancha, Albacete, Spain

**Keywords:** connectedness to nature, environmental identity, French context, scale validation, well-being

## Abstract

Connectedness to nature represents the relationship of the self with the natural environment and has been operationalized using different scales. One of the most systematically studied in the Anglo-Saxon context is the Connectedness to Nature Scale (CNS). In an attempt to study the psychometric properties of this instrument in a French-speaking context, three studies (Study 1 *n* = 204, Study 2 *n* = 153, and Study 3 *n* = 322) were carried out in France to provide evidence of the internal consistency of the CNS, as well as its convergent, discriminant, and predictive validity. Moreover, as anticipated, positive correlations between the CNS and the environmental identity and environmental concerns scales were observed. Based on factorial analyses of maximum likelihood and reliability, an improvement in the psychometric properties was identified by eliminating three items. Through confirmatory factor analysis, the factorial structure and the psychometric properties of the CNS French version were confirmed, as well as their significate regression prediction on eudaimonic wellbeing.

## Introduction

Connectedness to nature has been defined as a self-perceived relationship between the self and the natural environment (Schultz et al., 2004); it reflects a feeling of kinship and an affective individual experience of connection with nature (Mayer and Frantz, 2004). This concept is derived from studies on environmental concerns and has been proposed as being universal regarding the relationship between one’s self-image and nature, based on a biophilic disposition (Schultz et al., 2004; Mayer et al., 2009). In the same way, Kals and Ittner (2003; Kals et al., 1999) describe an emotional affinity with nature as an environmental identity (EID) indicator. They suggest that it is based on biophilia, a concept proposed by Wilson (1984) to express the feeling of an emotional link with the natural world, which means an inborn tendency to focus on life processes. This tendency is part of our genetic inheritance.

Schultz considers the valuation of the natural world as an extension of a person’s cognitive representation of him/herself, thus favoring the study of environmental concerns over environmental values as determinants of significant ecological change (Schultz et al., 2004). Schultz et al. (2004) have tackled research on the self-nature relationship by using different measures (the Nature in Self Scale – INS – and the Implicit Association Test – IAT). Another concept considers that in the building of a self-concept, nature and the self are not independent but linked, as the self-concept comes from a cognitive connection between nature and the self, facilitated by memories of oneself in nature (Thomashow, 1995; Schroeder, 2007; Olivos et al., 2013; Olivos and Clayton, 2017).

This is the concept of EID proposed by Clayton and Opotow (2003). In the studies carried out by these authors (Opotow, 1993, 1994; Opotow and Clayton, 1994), the implicit connection between human beings and nature corresponds to an axis ranging from people’s self-perception of superiority to plants and animals to a perception of identity that attributes the same rights to them as those of human beings.

Mayer and Frantz (2004) defined the connectedness to nature as an affective individual experience of connection with nature. To measure it, the authors presented the “Connectedness to Nature Scale” (CNS), probably the most studied scale (e.g., Frantz et al., 2005; Dutcher et al., 2007; Mayer et al., 2009; Nisbet et al., 2009; Perrin and Benassi, 2009; Brugger et al., 2011; Pasca et al., 2017). The authors’ analysis of the scale achieved an alpha score of 0.84 (Mayer and Frantz, 2004). Their results also showed, among other aspects, that the CNS correlates positively with biospheric concerns, the IAT-Nature and the INS, as well as with ecological behavior. In fact, it has been determined that connectedness to nature has a positive relationship with altruism, biospheric (Stern, 2000), and egobiocentric concerns (Olivos et al., 2011), environmental behaviors and, in a lesser way, life satisfaction. This dimension negatively correlates with conservatism (Mayer and Frantz, 2004) and non-environmental behaviors (Frantz et al., 2005), particularly when people have a more focused concern on themselves or a narcissistic personality.

These results allow the CNS study to be extended in relation to EID (Clayton, 2003) and environmental beliefs, such as anthropocentrism (ANT), “the dimension based on the instrumental value of the environment for human beings,” biospherism (BIO), “the dimension that values the environment for its own sake” and egobiocentrism (EGO), “the dimension that values the human being within nature as a whole” (Amérigo et al., 2007, pp. 98, 99). The theory of environmental beliefs gives a self-integration level in nature within two axes (Amérigo et al., 2012): the first one focuses on humans (EGO and ANT) and the second one focuses on nature (BIO). The relationship between the self and nature, characteristic of connectedness, should be closely linked to the kind of self-image and motivational beliefs that drive environmental behaviors. Thus, when we talk about the self as an EGO identity (e.g., Mayer and Frantz, 2004) or a metapersonal self (e.g., Olivos and Aragonés, 2014), it is similar to connectedness to nature, as this has been measured in recent years. Contact with natural environments have also been shown to have positive effects on well-being (Staats et al., 1997; Kaplan, 2001). It has indeed been observed that. It has been observed that connection to nature has a mediating effect in the increase of the positive emotional states (Mayer et al., 2009). Despite of these results, related to the called psychological well-being, their relation with subjective well-being remains scarcely studied (Olivos and Ernst, 2018).

Most of the instruments used for the study of environmental concerns originated in the Anglo-Saxon context and have gradually been adapted to other cultures and contexts, Spain and Portugal, especially. However, this has not yet been the case within the French speaking world for connectedness to nature, even though this kind of approach to studying human connection with nature represents one-third of the most recent research in this field (Ives et al., 2017). The growing interest of this dimension in the French-speaking countries requires the development of the validated and trustable tools to be able to study the links between connectedness to nature and the well-being and/or pro-ecological behaviors. We wonder whether the CNS (Mayer and Frantz, 2004) once adapted to the French language, keeps the same psychometric properties than the English version, which would help to measure the theoretical construct. Besides, France has an important tradition of studies in environmental psychology, who could benefit from the adaptation to its context of this scale. Our objective was thus to adapt and validate the Mayer and Frantz (2004) CNS within the French context as a contribution to studies about environmental concerns, which have become common in this cultural framework. This validation opens cultural perspectives as it contributes to the validation of connectedness to nature universal character, which is on the basis of this theory.

For this purpose, three studies were conducted to provide evidence of the internal consistency of the CNS, as well as its construct, convergent, discriminant, and predictive validity. The factorial structure of the scale was tested, in order to confirm these psychometric properties and the factorial structure of the CNS French version.

## Study 1

In this study, a descriptive analysis of the items and an exploratory factor analysis (EFA) were performed on a general population sample to identify the single factor structure of the CNS, following the proposal of Mayer and Frantz (2004).

### Method

#### Participants

The 204 participants were all living in a western French city (Nantes); women made up 72% of the sample. Average age *M* = 29 years (*SD* = 10.37). Regarding professional status, 60% were active, 6% unemployed, 1% retired, and 33% were students. This is about a convenience sample or group of volunteers. The margin of error with regard to the reference population is 6.8%. The rate of people in service is representative of the global population (60%), however, there is an over-representation of women (53% of the global population) and the average age is under the reference population (37 years old).

#### Material and Procedure

A self-administered questionnaire was used on paper-shaped, composed of the 14 items of the CNS and a five-point scale, ranging from “completely disagree” to “completely agree” to measure an affective individual experience of connection with nature (Mayer and Frantz, 2004). The scale was adapted to French using a two-way translation procedure (or back translation). This procedure consists in a native French-speaking translator with excellent English language skills translating the scale into French and a back translation of the previously obtained French version into English by an independent English speaking translator with excellent French language skills (Vallerand, 1989). The subjects were debriefed by telling them the aims of the study and their informed consent to participate was obtained. The mean time to complete the questionnaire was 10 min.

Reliability and factor analysis with SPSS 24 was carried out for a descriptive and psychometric study of the scale, which is the most usual procedure for establishing dimensionality of scales (Fabrigar et al., 1999; Embretson and Reise, 2000). Descriptive analyses (means, standard deviation, kurtosis, and asymmetry index) and reliability analyses (Cronbach’s alpha) were also performed.

### Results

#### Reliability and Descriptive Statistics

An EFA of the maximum likelihood following the procedure carried out by Mayer and Frantz (2004) and other studies of reference which analyses the psychometric properties of this scale (e.g., Perrin and Benassi, 2009; Tam, 2013; Olivos et al., 2014), forcing the extraction of a single factor explained 37% of the variances (KMO = 0.870; *p* < 0.001). The CNS showed a good level of internal reliability (α = 0.80). All the items had a positive load with values greater than 0.40 (see **Table 1**), except items 4 (fl = -0.13), 12 (fl = -0.17), and 14 (fl = -0.03), which were deleted according to the recommended load for samples between 200 and 250 participants (Hair et al., 1999).

**Table 1:** Exploratory factor analysis of principal components, reliability index and corresponding descriptive statistics of the CNS.

	Study 1 (*N* = 204)					
	*M*	*SD*	Asymmetry^a^	Kurtosis^b^	α^∗^	FL^c^
1. Je me sens souvent en union avec la nature qui m’entoure [I often feel a sense of oneness with the natural world around me]	3.45	0.98	-0.55	-0.11	0.78	0.63
2. Je pense à la nature comme à une communauté à laquelle j’appartiens [I think of the natural world as a community to which I belong]	3.37	1.01	-0.51	-0.24	0.77	0.74
3. Je reconnais et apprécie l’intelligence des autres êtres vivants [I recognize and appreciate the intelligence of other living organisms]	3.93	0.87	-1.07	1.75	0.78	0.59
4. Je me sens souvent déconnecté de la nature [I often feel disconnected from nature]	2.49	0.96	0.43	-0.26	0.82	-0.13
5. Quand je pense à ma vie, je m’imagine faisant partie d’un cycle de vie plus large [When I think of my life, I imagine myself to be part of a larger cyclical process of living]	3.32	1.08	-0.40	-0.46	0.77	0.58
6. Je me sens souvent un lien de parenté avec les animaux et les plantes [I often feel a kinship with animals and plants]	2.65	1.22	0.17	-0.99	0.76	0.72
7. Je me considère comme faisant partie de la Terre de la même façon qu’elle fait partie de moi [I feel as though I belong to the Earth as equally as it belongs to me]	3.03	1.18	-0.31	-0.80	0.76	0.67
8. Je comprends très bien comment mes actions ont un effet sur le monde naturel [I have a deep understanding of how my actions affect the natural world]	3.84	0.95	-1.07	1.25	0.79	0.38
9. Je me sens souvent comme faisant partie d’un écosystème plus large [I often feel part of the web of life]	3.60	1.00	-0.61	0.07	0.77	0.66
10. Je pense que tous les habitants de la Terre, humains et non humains, partagent une aaaforce vitale commune [I feel that all inhabitants of Earth, human and nonhuman, share a common ‘life force’]	3.19	1.06	-0.42	-0.28	0.78	0.54
11. Tout comme l’arbre fait partie de la forêt, je me sens comme faisant partie de la nature [Like a tree can be part of a forest, I feel embedded within the broader natural world]	3.36	1.01	-0.50	-0.13	0.76	0.77
12. Lorsque je pense à ma place sur Terre, je me considère comme faisant partie de l’espèce supérieure [When I think of my place on Earth, I consider myself to be a top member of a hierarchy that exists in nature]	2.30	1.06	0.62	-0.24	0.83	-0.17
13. J’ai souvent l’impression que je ne suis qu’ une petite partie de la nature qui m’entoure et que je ne suis pas plus important que l’herbe sur le sol ou les oiseaux dans les arbres [I often feel like I am only a small part of the natural world around me and that I am no more important than the grass on the ground or the birds in the trees]	3.50	1.13	-0.44	-0.71	0.78	0.60
14. Mon bien-être personnel est indépendant du bien-être du monde naturel [My personal welfare is independent of the welfare of the natural world]	2.62	1.01	0.051	-0.82	0.82	-0.03

*^a^ Standard error asymmetry = 0.170; ^b^ Standard error kurtosis = 0.339; ^∗^Cronbach’s alpha if items are deleted; ^c^ Forced extraction of a single factor (Test of goodness of fit: χ^*2*^= 208.639; *df* = 77, *p* < 0.00. χ^*2*^/*df* = 2.71).*

## Study 2

The objective of this second study was to confirm, on a second sample of the general population, the single factor structure of the CNS. In addition, we wanted to assess the internal consistency and validity of the CNS through convergent validity by correlating its results to the Environmental Identity Scale (EID) as proposed in the literature (Brugger et al., 2011; Olivos et al., 2013; Olivos and Clayton, 2017). A positive correlation was expected regarding the connectedness and EID measures.

### Method

#### Participants

In this study, 153 people from the general population participated voluntarily and anonymously (7.9% margin of error with regard to the reference population). Of these, 24.2% were students, 54.9% had a professional activity, and 7% were unemployed. Women made up 58.8% of the sample. Regarding their age, 63.4% were between 18 and 29 years. 26.1% between 30 and 49 years and 10.5% were more than 50 years old (*M* = 30.5; *SD* = 10.75).

#### Material and Procedure

A self-administered questionnaire was used, similar to the questionnaire of Study 1, composed by the CNS and EID. The subjects were debriefed by telling them the aims of the study and their informed consent to participate was obtained. The administration of the scales took about 15 min. The CNS consisted of 11 items (three items were eliminated, 4, 12, and 14, according to the results of the EFA of Study 1) on a five-point scale, ranging from “completely disagree” to “completely agree.” The EID (Clayton, 2003) consisted of 24 items on a five-point scale, ranging from “completely disagree” to “completely agree,” to measure the relationship between self and nature.

A confirmatory factor analysis (CFA) was conducted to validate the factorial structure with R. We kept the 11 items that had acceptable indicators in the CFA. The maximum likelihood method was selected to test the model. To assess the fit of the model, χ^2^, the Comparative Fit Index (CFI), the Tucker Lewis Index (TLI), the Goodness of Fit Index (GFI), the Standardized Root Mean Square Residual (SRMR), and the Root Mean Square Error of Approximation (RMSEA) were examined. Lastly, the saturation coefficients among items and the latent variables were examined. A value superior to 0.90 for the CFI, GFI, and the TLI is sufficient (Tucker and Lewis, 1973; Bentler, 1992; Schumacher and Lomax, 1996). A RMSEA and SRMR lower than 0.08 (Browne and Cudeck, 1993; MacCallum et al., 1996; Pui-Wa and Qiong, 2007) is admitted. Concerning the use of χ^2^, it is possible that the tested model does not fit the data correctly, but that χ^2^ accepts it because of the size of the sample (Pui-Wa and Qiong, 2007). For this reason, Wheaton et al. (1977) suggest that a relative chi-squared (χ^2^/*df* or CMIN/*df*) is also computed. A χ^2^/*df* ratio < 3.00 represents a correct fit.

### Results

#### CFA and Reliability Analysis

The reliability of the scale was estimated by calculating the Cronbach’s alpha coefficient and composite reliability (CR, Raykov, 1997) for CNS. The CNS showed a good level of internal reliability (α = 0.85; CR = 0.88), as did the EID (α = 0.93). The tested model fitted the data correctly, except TLI, which is lightly under the expected threshold [RMSEA _(90%CI)_ = 0.095 _(0.07-0.12)_; CFI = 0.909; TLI = 0.887; GFI = 0.923; SRMR = 0.052]. Because of a significant χ^2^ (*p* < 0.001), we examined the χ^2^/*df* ratio. With a value of 2.35, it can be considered correct.

#### Correlation

The correlation between the CNS and EID (*r* = 0.763; *p* < 0.001) was positive and statistically significant, indicating the convergent validity of the CNS.

## Study 3

This study aimed first to confirm the single factorial structure of the CNS in a second sample. In addition, we sought to assess the internal consistency (Cronbach’s alpha) and validity of the CNS: for convergent validity, CNS would correlate positively with the EID and EGO (Amérigo et al., 2007); for discriminant validity, the CNS would correlate negatively with ANT (Amérigo et al., 2007); for predictive validity, the CNS would predict scores of the wellbeing scale (MHC-SF, Keyes, 2009) as well as the frequency of contact with nature.

### Method

#### Participants

In this study, 322 participants were distributed into two samples. The first sample (A) was composed of 267 students from a French university; 85% were women and the average age was *M* = 19.60 (*SD* = 3.75) years. The second sample (B) was 55 students from the same university, who completed the instruments twice; 61.8% were women and the average age was *M* = 22.24 (*SD* = 5.04) years.

#### Material and Procedure

The instrument used for both samples was a self-administered questionnaire composed of the following scales: the CNS (Mayer and Frantz, 2004) and the EID Scale (Clayton, 2003), the same scales as in Study 2; two scales to measure environmental concerns, ANT (to assess the convergent validity of the CNS) and EGO (to assess the convergent validity), in the version of Amérigo et al. (2007), composed of five items on a five-point scale, ranging from “completely disagree” to “completely agree”; the Mental Health Continuum Short Form (MHC-SF, Keyes, 2009), applied in similar investigations and obtaining good psychometric indicators (Aragonés et al., 2011), which consists of 14 items measuring Hedonic Wellbeing (MHC.H – pleasure-related or experienced emotions) and Eudaimonic Wellbeing (MHC.E – related to psychological development and personal growth), and a whole general wellbeing index; lastly, the variable “contact with nature” was operationalized with three modalities (never, occasionally, and frequently) of activities in natural places (e.g., “ Do you realize activities in touch with nature during your spare time, like picnics, walks on the beach or in a park, hiking, etc.?”).

All these scales were adapted to French using the two-way translation procedure. The subjects were debriefed by telling them the aims of the study and their informed consent to participate was obtained. Each application lasted on average 20 min and was carried out by both samples at the beginning of a class. Sample B completed the questionnaire again 2 weeks after the first time (for the test–retest reliability).

#### Analysis

Data analysis was carried out for descriptive (means, standard deviation, kurtosis, and asymmetry index) and psychometric (reliability and factor analysis) studies of the scale, including test–retest for the CNS and EID with sample B. Correlations and mean difference analyses were performed to test convergent (EID and EGO) and discriminant (ANT) validity. A regression analysis also tested the predictive validity of the MHC-SF scale, the same as the correlation between the CNS and contact with nature. A CFA was used to verify the factorial structure of the CNS as in Study 2.

### Results

#### CFA of the CNS and Reliability Analysis

A CFA with sample A (*n* = 267) was carried out. The tested model fitted the data correctly, except TLI, which is lightly under the expected threshold [RMSEA_(90%*CI*)_ = 0.071 _(0.05-0.08)_; CFI = 0.912; TLI = 0.890; GFI = 0.902; SRMR = 0.051). Because of a significant χ^2^ (*p* < 0.001), we examined the χ^2^/*df* ratio (115.595/44). With a value of 2.62, it can be considered correct. We observed that the indices were correct and improved compared to Study 2, especially the RMSEA that was correct this time (<0.08).

All scales reached a good internal reliability score in sample A (see **Table 2**).

**Table 2:** Descriptive statistics and reliability (sample A, *n* = 267).

	*M*	*SD*	Asymmetry	Kurtosis	α
CNS	3.34	0.55	-0.033^a^	-0.269^b^	0.800
EID	3.26	0.58	-0.230	-0.275	0.904
EGO	3.84	0.71	-0.550	-0.119	0.792
ANT	2.25	0.75	0.629	0.428	0.749
MHC	3.30	0.47	-0.518	0.599	0.797
MHC.H	3.77	0.60	-0.809	1.696	0.730
MHC.E	3.17	0.49	-0.432	0.461	0.743

*^a^ Standard error asymmetry = 0.136; ^b^ Standard error kurtosis = 0.271; CNS, Connectedness to Nature Scale; EID, environmental identity; EGO, egobiocentrism; ANT, anthropocentrism; MHC, Mental Health Continuum, Short Form; MHC-H, Hedonic; MHC-E, Eudaimonic.*

The test–retest analysis with answers of sample B (see **Table 3**) showed a good level of reliability too for the CNS [*r* = 0.774; *p* < 0.001; *t*(54) = 0.2160; *p* = 0.830] and EID [*r* = 0.865; *p* < 0.001; *t*(54) = -1.30; *p* = 0.198].

**Table 3:** Test–retest reliability of the CNS and EID (sample B, *n* = 55).

	*M*	*r*	*t*	*p*
CNS	3.33	0.774^∗∗∗^	0.216	0.830
CNS - POST	3.32			
EID	3.25	0.865^∗∗∗^	-1.303	0.198
EID - POST	3.31			

*^∗^*p* < 0.05; ^∗∗^*p* < 0.01; ^∗∗∗^*p* < 0.001.*

#### Correlations and Regression

To provide support for the convergent and discriminant validity of the CNS scale, its average score was correlated with the scores of the other complementary measures such as the EID and MHC, the two scales of environmental concerns (ANT and EGO) and the measure of frequency of contact with nature (CN). The results are presented in **Table 4**.

**Table 4:** Correlation between variables for convergent and divergent validity (sample A, *n* = 267).

	1	2	3	4	5	6	7
1. CNS	–						
2. EID	0.701^∗∗^	–					
3. EGO	0.596^∗∗^	0.714^∗∗^	–				
4. ANT	-0.234^∗∗^	-0.206^∗∗^	-0.294^∗∗^	–			
5. MHC	0.095	0.079	0.063	0.085	–		
6. MHC-H	0.014	-0.034	-0.036	0.037	0.719^∗∗^	–	
7. MHC-E	0.110^∗^	0.108	0.088	0.091	0.973^∗∗^	0.539^∗∗^	–
8. CN	0.348^∗∗^	0.499^∗∗^	0.392^∗∗^	-0.126^∗^	0.085	0.067	0.081

*^∗^*p* < 0.05; ^∗∗^*p* < 0.01, ^∗∗∗^*p* < 0.001 (bilateral). CN, contact with nature.*

The correlations between the CNS and EID were positive and statistically significant, thus consistent with what was expected. Furthermore, the CNS correlated positively with EGO and negatively with ANT, showing the expected relationships with these environmental concerns. The correlations were weak and not significant with Wellbeing but, as expected, positive and significant with the sub-dimension of MHC.E. The regression analysis confirmed the predictability of MHC.E from the CNS and EID (see **Table 5**).

**Table 5:** Regression analysis to predict MHC-E from the CNS and EID.

Step	Variable	*R*	*ΔR*^2^	*F*	β
1	CNS	0.702	0.493	257.511^∗∗^	0.702^∗∗^
2	CNS	0.710	0.504	6.191^∗∗^	0.607^∗∗^
	EID				0.143^∗^

*^∗^*p* < 0.05; ^∗∗^*p* < 0.01, ^∗∗∗^*p* < 0.001.*

Finally, the correlation with the frequency of contact with nature was statistically significant and positive (*r* = 0.348, *p* < 0.001). Moreover, the mean difference analysis in the score of the CNS by contact with nature showed statistically significant results (*t* = 4.431; *df* = 320; *p* < 0.001), suggesting that participants who had taken part in activities involving contact with nature experienced higher levels of connectedness to nature (*M* = 3.41; *SD* = 0.54) than participants who had not (*M* = 3.09; *SD* = 0.52).

The results indicate that the CNS has good psychometric properties, which improved after some items were deleted (items 4, 12, and 14). The coherent correlations between the measures of connectedness and environmental concerns and EID suggest that people connected to nature value the positive effects of each personal experience with nature, within which they feel explicitly included, and do not subordinate it to human needs.

## Discussion

These studies have enabled the verification of the internal positive consistency of the CNS, in the same way as the authors of the original scale in other investigations (Mayer and Frantz, 2004; Frantz et al., 2005; Mayer et al., 2009), yet within a psychometrically acceptable range (Cortina, 1993; George and Mallery, 2003). This scale is evidently stable and the comparison of its scores with EID and environmental concerns (ANT, EGO) shows evidence of its convergent and discriminant validity, as well as providing an opportunity to propose conceptual questions that might guide new research concerning connectedness to nature in French-speaking contexts, where this subject is gaining interest.

The specific results suggested the elimination of items 4, 12, and 14 (“I often feel disconnected from nature,” “When I think of my place on Earth, I consider myself to be a top member of a hierarchy that exists in nature” and “My personal welfare is independent of the welfare of the natural world”; Mayer and Frantz, 2004, p. 513) because of their lower loading weight (Hair et al., 1999) and because the consistency markers of the scale improved after the elimination of these items. The CFA showed that, without these items, the scale gave good marks of reliability as well as a good fit of its overall factor structure. In the same way as other psychometric studies, which have suggested the advantage of deleting some items in specific cultural contexts (Olivos et al., 2011; Pasca et al., 2017), this result demonstrates the interest of proposing a new version of this scale, in order to obtain the best psychometric qualities in the French version.

As expected, the correlation between the CNS and EID was also positive, contributing to the validity of both measures. However, these results should be analyzed with caution. Despite the fact that the EID has obtained higher reliability values than in this investigation (Clayton, 2003), more studies have been published on the EID that cast doubt on its psychometric properties and factorial structure (Olivos and Aragonés, 2011; Clayton, 2012). Furthermore, despite both scales referring to a type of relationship of identification with the natural environment, in the case of connectedness their authors proposed that there is an underlying idea of a biological disposition favorable to nature (biophilia), and thus of universal occurrence. Other studies could be lead in order to verify this hypothesis within the French context, such as for example the biological disposition of connectedness, which suggests a restoring effect of natural environments (Mayer et al., 2009).

A significant correlation was observed between the scores of the CNS and those of environmental concerns. In the case of ANT, the correlation was negative, as anticipated, because an instrumental valuation of nature is clearly opposed to the idea suggested in connectedness. In the case of EGO, the correlation was positive, which is coherent with connectedness due to the valuation it makes of the relationship between the human being and nature as a whole.

Positive and significant correlations with the frequency of contact with nature indicated that the more connected people feel to nature, the more they will try to keep in contact with it. Unfortunately, the disappointing results of the relationship with wellbeing prevent us from concluding that this connection with nature involves a feeling of wellbeing. However, the positive and significant although weak correlation with Eudemonic Wellbeing is an important topic for environmental psychology research. Even if hedonic experiences have been more frequently studied, the eudemonic dimension of wellbeing is more closely linked to the development of positive and complex identities. Besides, this eudemonic dimension is linked to subjective connections with nature (Arnocky et al., 2007; Leary et al., 2008; Clayton, 2012; Ryff and Singer, 2013; Olivos and Aragonés, 2014; Olivos and Ernst, 2018).

On the basis of this study, it can be concluded that the CNS is a valid and reliable measure of connectedness, useful for research in psychology concerning the processes of environmental concerns, the restoring effect of natural environments, the perception of natural risks, etc., as well as being a valid tool for the study of connectedness in a French-speaking context. This version of 11 items proposed at the end of the study, could be very well integrated to the analysis of the relation between connectedness to nature with other dimensions as well-being, environmental concerns and even perception of natural risks. Nevertheless, some limits must be underlined. Actually, participants are not representative of French population, even if the margins of error of the sampling are relatively low. On the same way, marked cultural differences between French-speaking countries should also be taken into account during future applications. Actually, the sharing of a common language does not cancel the cultural diversity in the meaning attributed to some built, being able to make results vary. Anyway, this psychometric French speaking version of CNS, allows to initiate a systematic research for its adaptation in other French-speaking regions.

## Ethics Statement

These studies were approved by the ethical board of the Psychology Faculty of University of Nantes with written informed consent from all subjects.

## Author Contributions

ON: work conception, research design, data collection, data analysis, data interpretation, paper arrangement and revision, writing, and submission. PO: research design, data analysis, data interpretation, paper arrangement and revision, and writing. GFB: data collection, research design, data interpretation, paper arrangement and revision, and writing.

## Conflict of Interest Statement

The authors declare that the research was conducted in the absence of any commercial or financial relationships that could be construed as a potential conflict of interest.
